# Wastewater surveillance for COVID-19 in shelters: A creative strategy for a complex setting

**DOI:** 10.14745/ccdr.v50i12a07

**Published:** 2024-01-01

**Authors:** Chalani Ranasinghe, Stefan Baral, Rebecca Stuart, Claire Oswald, Sharon Straus, Amir Tehrani, Kimberley Gilbride, Princilla Agyemang, Aaron Orkin

**Affiliations:** 1 Dalla Lana School of Public Health, University of Toronto, Toronto, ON; 2 Department of Family and Community Medicine, Faculty of Medicine, University of Toronto, Toronto, ON; 3 Inner City Health Associates, Toronto, ON; 4 Knowledge Translation Program, Unity Health Toronto, Toronto, ON; 5 Toronto Public Health, Toronto, ON; 6 Department of Geography and Environmental Studies, Toronto Metropolitan University, Toronto, ON; 7 Department of Medicine, University of Toronto, Toronto, ON; 8 Department of Chemistry and Biology, Toronto Metropolitan University, Toronto, ON; 9 MAP Centre for Urban Health Solutions, Unity Health Toronto, Toronto, ON

**Keywords:** vulnerable populations, wastewater-based epidemiological monitoring, public health surveillance, COVID-19

## Abstract

People experiencing homelessness experience disproportionate rates of morbidity and mortality from coronavirus disease 2019 (COVID-19) compared to the general population and shelters for people experiencing homelessness are a major contributing factor to these negative outcomes. As a result of their unique structure, population and physical space, these settings pose several challenges to the prevention of COVID-19 infection that are not adequately addressed by conventional non-pharmaceutical public health interventions. Wastewater surveillance for COVID-19 is a viable strategy for health protection in shelters due to its ability to meet these unique challenges. Its passive nature does not depend on individual health-seeking behaviours, and it can provide useful epidemiological information early on in an outbreak setting. In this commentary, the authors examine a recent application of wastewater surveillance of COVID-19 in a men’s shelter in Toronto. Further applications of wastewater surveillance for other infectious diseases of concern in shelters are proposed, and the need for the development of ethical frameworks governing the use of this technology is discussed.

## Introduction

As of June 2023, Canada had reported over four million cases of coronavirus disease 2019 (COVID-19) and 40,000 COVID-19-related deaths ((1)). Although the impacts of COVID-19 were widespread, there were significant and sustained disparities in outcomes across Canada ((2)). People experiencing homelessness (PEH), a population estimated at 235,000 in Canada in a given year ((3)), were disproportionately harmed by COVID-19. People experiencing homelessness in Canada face a greater burden of severe acute respiratory syndrome coronavirus 2 (SARS-CoV-2) infections, as well as increased rates of hospitalizations, intensive care unit admissions and mortality from COVID-19 ((4)). People experiencing homelessness are affected by several inequities, collectively increasing their risk of COVID-19-related morbidity and mortality, including high rates of chronic illness and decreased access to healthcare services ((5,6)). Non-pharmaceutical interventions including physical distancing, screening for symptoms, testing and isolation are difficult to implement in a community burdened by mental health and substance use, and with existing distrust in healthcare institutions ((7,8)).

The physical context in which PEH live, interact and access resources can exacerbate many of these risks. Shelters for PEH feature high population density and rapid turnover, client marginalization and poverty, poor ventilation, lack of access to optimal hygiene, insufficient infection control and other regulatory protections, and limited staff training and resources, all of which increase the risk of transmission of COVID-19 and other respiratory diseases ((9)). Although guidelines for the control of COVID-19 in shelters have been developed and recommended by public health organizations during earlier phases of the pandemic ((10)), shelter service providers described feelings of uncertainty and powerlessness given limited resources in the support of PEH during the pandemic ((11)). These factors together contribute to the increased prevalence of COVID-19 in shelters relative to other settings ((12)). Shelters represent a specific setting, serving a unique population, requiring targeted strategies to prevent, identify and respond to COVID-19 and other communicable conditions.

Wastewater surveillance is a disease surveillance strategy in which sewage samples are routinely tested to identify the presence of, and quantify trends in, pathogens of interest. Wastewater surveillance has been used for the detection of poliovirus and human enteroviruses in communities ((13,14)). In recent years it has emerged as a tool to monitor SARS-CoV-2 and has been employed in high-risk settings such as correctional facilities and long-term care homes ((15–17)). Akingbola *et al.* described the successful implementation of a wastewater surveillance strategy in a men’s shelter in Toronto, Ontario, where this approach facilitated the early detection of an outbreak and prompted measures to prevent further transmission in this setting ((18)). By testing for and monitoring communicable diseases at the community or facility level—rather than the individual case or patient level—wastewater surveillance combines elements of communicable disease and environmental health strategies. Like air or water quality surveillance systems, wastewater monitoring seeks to identify threats to public health and inform appropriate responses regardless of whether they have already elicited clinically identifiable morbidity. These kinds of strategies are needed to address and reduce the burden of communicable conditions in congregate settings such as shelters.

**Figure 1:**
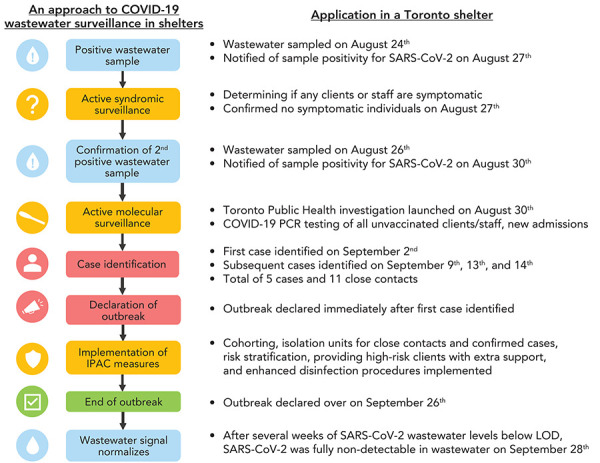
Schematic of an approach to COVID-19 wastewater surveillance in shelters Abbreviations: COVID-19, coronavirus disease 2019; IPAC, infection prevention and control; LOD, level of detection; PCR, polymerase chain reaction; SARS-CoV-2, severe acute respiratory syndrome coronavirus 2

Monitoring wastewater for infectious diseases addresses some of the observed challenges in mitigating COVID-19 risks in shelters serving PEH. In many instances, positive signals in wastewater samples can be detected early in the disease course prior to symptom onset or in asymptomatic infections. This creates enhanced situational awareness and provides useful lead time for response, including earlier outbreak control. Wastewater monitoring can be part of a rapid response strategy in which a positive signal triggers immediate implementation of heightened infection protection and control measures, such as syndromic and molecular surveillance, enhanced cleaning procedures and support of at-risk clientele ((18)) (**Figure 1**).

Wastewater surveillance is passive and does not rely on individual health-seeking behaviours ((19)), which is a benefit in a population that experiences decreased access to healthcare and may be reticent to disclose communicable symptoms in congregate settings. Similarly, as access to polymerase chain reaction testing continues to decrease, with rapid testing becoming more prevalent, wastewater surveillance provides an additional tool for ongoing facility- and community-level monitoring to track trends and inform action and policy ((20)). Wastewater surveillance for shelters has since been expanded to include other pathogens that PEH are at risk of contracting, including influenza and respiratory syncytial virus, similar to other community settings ((21,22)). Future applications in shelters can be used to monitor critical pathogens such as hepatitis A virus ((23)).

In the authors’ experience of wastewater surveillance in Toronto, the marginal cost per sample was approximately CA$105, with additional costs incurred for additional testing sites, need for additional laboratory personnel and logistical factors. An economic analysis of wastewater surveillance in Japan favoured the use of wastewater surveillance over rapid antigen tests at single institutions, particularly at lower incidences of COVID-19 ((24)), although the generalizability of this study to a Canadian context is limited. An economic analysis of wastewater surveillance and rapid antigen testing in a Canadian context would be valuable.

Wastewater testing has prompted legitimate ethical discussions and the need for sound ethical frameworks to govern its use ((25)). In the case of small-scale, near-source testing, this strategy can be context specific to meet the needs and affirm the rights of the population being served. This proximity necessitates that shelter service providers and people with lived experience of homelessness be engaged in guiding data collection, usage and responses in wastewater testing. Collaboration with partners in shelters, healthcare, public health, environmental services and ethical bodies can make shelter-based wastewater surveillance both effective and culturally appropriate.

Shelters are an essential resource and safety measure for PEH, but also challenge efforts to protect residents from health threats, including communicable diseases. Shelters, while necessary to provide accommodations and support for PEH, cannot replace accessible, affordable housing for all. Wastewater surveillance may serve to decrease unnecessary morbidity and mortality associated with homelessness alongside measures to end homelessness itself ((8,26)).

## Conclusion

The COVID-19 pandemic has revealed that conventional approaches to communicable disease surveillance, case finding, outbreak response and health protection will continue to yield sustained inequities in exposure and access to preventive interventions. Innovative, community-responsive strategies like wastewater surveillance offer alternative and assertive approaches to redress these inequities. Leadership in this area by the Public Health Agency of Canada’s National Wastewater Surveillance Program has fostered national support and collaboration for the use wastewater surveillance. Further support and meaningful intersectoral engagement from public health agencies, congregate settings and networks, water and sanitation systems, and academic centres will be necessary to steward the sustainable, effective implementation of this intervention. As communities transition into COVID-19 recovery, we face the threat that innovations developed in the context of crises might be cast aside as unwarranted or unworthy of sustained investments. At this juncture, we can and should invest in long-term programs including improved surveillance and service delivery to better address health risks faced by the most marginalized members of our community, or we can risk having learnt little from COVID-19.
